# Immunological Properties of Neoplastic Neural Tissues

**DOI:** 10.1038/bjc.1971.86

**Published:** 1971-12

**Authors:** H. R. Wickremesinghe, P. O. Yates

## Abstract

**Images:**


### IMMUNOLOGICAL PROPERTIES OF NEOPLASTIC NEURAL TISSUES

H. R. WICKREMESINGHE* AND P. O. YATES†

*From the Department of Neuropathology, University of Manchester, Manchester, M13 9PL*

Received for publication August 18, 1971

**SUMMARY.—The results are presented of the examination of 28 neoplasms from the nervous system for an organ-specific antigenic material demonstrated in glial cells and myelin, using gel diffusion, immunocytotoxicity and immunofluorescence techniques.**

**The antigenic material was demonstrable in benign gliomas and in those of low-grade malignancy, but not in the more malignant gliomas and non-gliomatous neoplasia.**

**The implications of the loss of specific antigenic material with reference to cell structure and function, and some fallacies in the interpretation of the significance of tissue specific antigen in carcinogenesis are discussed.**

THE presence of an immunologically specific material in normal astrocytes, oligodendrocytes and myelin was demonstrated by the authors and reported in a separate publication (Wickremesinghe and Yates, 1971).

The antiserum which was prepared for the study was capable of demonstrating the presence of this specific material in tissues, using the following immunological techniques:

1. Double diffusion in agar gel;
2. Cytotoxic effect of antiserum on living tissue;
3. Localization of antigen using fluorescent antiglobulin.

The present paper reports a complementary aspect of the study: the distribution of the immunologically specific material in various neoplasms of neural origin.

### MATERIAL AND METHODS

A detailed description of the material and methods used has been reported in the publication dealing with the findings in normal brain tissue (Wickremesinghe and Yates, 1971). A summary of the relevant description of material and methods is included in this section.

*Tissue.*—Normal brain, kidney, liver, thyroid, pancreas, heart muscle and nerve were obtained from fresh autopsy material within 12 to 18 hours of death.

Fresh neoplastic tissue (as well as some normal brain tissue) was obtained from surgical specimens within an hour or two of removal.

* Present address of Dr. Wickremesinghe: Department of Pathology, General Hospital, Colombo, Ceylon.

† Requests for reprints, and correspondence, to be addressed to: Professor P. O. Yates, Department of Neuropathology, University of Manchester, Manchester M13 9PL.

*Methods.*—Tissue homogenates for use as antigen were prepared by mincing frozen blocks of the tissue on a microtome set to cut at 2·5 $\mu$ (Tee, Wang and Watkins, 1964). The minced tissue was deposited into phosphate buffered saline (1·5 ml. of phosphate buffered saline per g. of minced tissue).

In preparing brain tissue homogenate, representative blocks were taken from cerebral and cerebellar cortex and white matter and basal ganglia.

Care was taken to exclude normal brain tissue in preparing neoplastic tissue homogenates, by examining histological sections of the material used.

*Antiserum.*—Antiserum to normal human brain tissue was prepared in rabbits. A pre-immunization sample of blood was obtained from each of the three rabbits used. Thereafter, 0·4 ml. of the homogenized brain tissue with complete Freund's adjuvant was injected intradermally into 4 widely separated sites on the shaved back of each rabbit. These injections of antigen were repeated at weekly intervals for several months.

Blood for preparation of the antiserum was collected under sterile conditions from the marginal ear vein of the rabbit.

Absorptions of antisera: The following absorptions were carried out:

(a) Absorption of sera with homogenates of liver, thyroid, kidney and pancreas;
(b) Absorption of sera with homogenates of normal brain tissue.

*Decomplementing of sera.*—This was achieved by heating the sera at 56° C. for 30 minutes in a water bath.

*Immunodiffusion in agar gel.*—The agar double diffusion technique of Ouchterlony (1948, 1964) and Elek (1948) was adapted.

Seven ml. of melted 0·8% agar (Noble Agar, Difco) in phosphate buffered saline were allowed to spread as a thin film of uniform thickness on a glass microscope slide 38 × 75 mm., fitted into a rectangular Perspex chamber. Wells were punched out of the solidified agar, using sets of cylindrical cutters fixed on a metal plate.

The wells were filled with the same amount of reactants (antiserum or tissue homogenate). The slides were then kept on moist filter paper in a closed container for 4 days, immersed for 24 hours in phosphate buffered saline, followed by immersion in water for a like period, dried, and stained with Amido Black.

The arrangement of wells in agar gel shown in Fig. 1 was used to screen samples of neoplastic tissue homogenate for their ability to give precipitin lines.

If precipitin lines were detected, the arrangement of wells shown in Fig. 3 was used to determine whether or not there was confluence with the precipitin line specific for brain tissue.

### *Immunofluorescence studies on cryostat sections*

The sandwich technique (Weller and Coons, 1954) was adapted for tissue sections and explant cultures. A drop of the prepared antiserum was spread over tissue section or explant culture and incubated at 37° C. for half an hour. The preparation then was washed for a total period of 10 minutes in three changes of phosphate buffered saline with " micro-agitation ". (This was obtained by keeping the slides on the cover of a functioning centrifuge.) Thereafter a drop of fluorescein-labelled rabbit globulin antiglobulin was spread over the preparation, incubated again at 37° C. for half an hour, and washed in three changes of phosphate buffered saline as before. The preparation was mounted in 50% glycerin in phosphate buffered saline.

These were examined with a Leitz Ortholux microscope equipped for phase contrast and fluorescence microscopy. The localization of the antiglobulin, and therefore the antibodies, was shown by its bright green fluorescence in u.v. light.

Modifications introduced to minimize non-specific staining:

(a) The fluorescein labelled antiglobulin used in the experiments (Bacto FA rabbit globulin antiglobulin-Goat) was absorbed with washed packed wet tissue homogenate of liver to selectively remove fluorescein conjugated serum proteins other than the globulin, since these could cause non-specific staining (Kaplan, 1958). Such absorption also removed unreacted fluorescent material (Nairn, 1964).

(b) Cryostat sections used in these investigations were stored for 48 hours at −20° C. before use. Mayersbach (1959) has shown that non-specific protein binding is decreased or abolished in sections which have been stored for several days.

*Tissue culture*

(a) Explant culture. Selected fragments of tissue were rinsed in medium 199 (Morgan, Merton and Parker, 1950), and cut into fragments 2 mm. or less in diameter. A small drop of chicken plasma and thrombin was mixed on the cover-slip, and spread over it. The three fragments were then evenly spaced on the coverslip, before the plasma could clot. The coverslip was introduced into the rectangular well of a Leighton tube (Leighton, 1954) and 1 ml. of culture medium added into the well. A number of tissue fragments were also explanted on bare glass coverslips without the use of plasma clot.

Cultures in Leighton tubes were observed *in situ* using a Leitz inverted micro-scope. After adequate growth had been established, detailed observations by phase contrast microscopy were carried out by sealing the coverslip cultures with hot wax on thin perspex chambers with a rectangular well containing culture medium. It was possible to maintain the cultures at a temperature between 36° and 38° C. by enclosing the microscope lamp housing and a thermostatically controlled heating coil, under a polythene bag with arm holes to permit manipulation of the microscope.

(b) Organ culture. Tissue in organ culture thrives best at or near the air-medium interface. We adopted the following simple technique, for short-term organ culture.

Three or four fragments of tissue, each about 3 mm. in diameter, were supported on lens paper carried on a rectangular piece of fine stainless steel mesh, 30 × 10 mm., which was slipped into a test tube, and sufficient culture medium added to just cover the mesh, with the tube tilted at an angle of about 15°. The tube was stoppered and incubated at 37° C.

(c) Culture medium. Medium 199 supplemented with 20% horse serum was used. Any complement in the latter was inactivated by heating it at 56° C. for 30 minutes. Penicillin (50 units per ml.) and streptomycin (50 mg. per ml.) were added to the culture medium. The effect of normal rabbit serum, or antiserum on the cultures was observed by adding it (with and without complement) to the culture medium.

The initial pH of the culture medium was adjusted to 7·2 by bubbling carbon dioxide.

### *Critical Evaluation of Methods*

Two of the methods used—immunocytotoxicity and immunofluorescence—can give equivocal results, because non-specific reactions are not uncommon.

A critical evaluation of the modifications and techniques and the controls we used to distinguish between specific and non-specific reactions, is given in the publication which reported our findings in normal brain tissue (Wickremesinghe and Yates, 1971). These same modifications and controls were adopted in the observations reported in the present paper.

### RESULTS

### *Double diffusion in Agar gel*

Normal brain tissue gave several precipitin lines when reacted with unabsorbed antiserum (Fig. 1). Our observations on the reaction of unabsorbed and absorbed antiserum with a variety of normal tissues has shown that brain tissue gives a precipitin line (marked " B " in the figure) which is not given by any other tissue (Fig. 2) and which is not eliminated by absorption of antiserum with any tissue other than brain (Wickremesinghe and Yates, 1971). This " B " line is therefore organ specific for brain tissue. All the precipitin lines other than the " B " line are non-specific in that they are eliminated by absorption by other tissues as well as brain tissue (Fig. 3).

---

### EXPLANATION OF PLATES

FIG. 1.—" A " contained unabsorbed antiserum. Peripheral wells 1 to 6 contained brain tissue homogenate. The arrow indicates the " B " precipitin line specific for brain tissue.

FIG. 2.—" A " contained unabsorbed antiserum. Peripheral wells 1 to 6 contained liver tissue homogenate. The precipitin lines (arrows) correspond to the inner group given by brain tissue homogenate. The " B " line is absent.

FIG. 3.—" A " contained antiserum absorbed with a mixed homogenate of liver, kidney and thyroid tissue. 2, 4, and 6 contained brain tissue homogenates. 1, 3 and 5 contained kidney, thyroid and liver tissue homogenates respectively.

FIG. 4.—" A " contained antiserum absorbed with a mixed tissue homogenate of liver, kidney and thyroid. 1 contained tissue homogenate from an oligodendroglioma. 2, 4 and 6 contained normal brain tissue homogenate. 3 and 5 contained tissue homogenate from 2 glioblastomas. The precipitin lines of brain tissue is confluent with that of the oligodendroglioma. The glioblastomas do not give a precipitin line.

FIG. 5.—The appearance of astrocytic neoplastic cells before (5a) and after (5b) the addition of antiserum and complement to the culture medium.

FIG. 6.—Normal brain tissue treated with immune serum followed by fluorescein labelled antiglobulin. Staining of the perikarya gives sharp definition of the unstained nuclei.

FIG. 7.—Normal brain tissue treated with normal rabbit serum followed by fluorescein labelled antiglobulin. The nuclei are not defined, as perikarya are not stained. There is non-specific background staining of myelin sheaths.

FIG. 8.—Cells of oligodendroglioma showing intense immunofluorescent staining of perikarya in cryostat section.

FIG. 9.—Astrocytic neoplastic cells in culture showing irregular immunofluorescent staining of perikarya and processes.

FIG. 10.—Astrocytic neoplastic cells in culture, showing intense focal immunofluorescent staining of perikarya, and absence of staining of processes.

FIG. 11.—Oligodendroglioma cells in culture showing marked immunofluorescent staining of perikarya and processes.

Wickremesinghe and Yates.

5a 5b 6 7

Wickremesinghe and Yates.

Wickremesinghe and Yates.

In all gel diffusion observations carried out in the present study, on neoplastic tissue, antiserum was used which after adequate absorption with a mixed homogenate of liver thyroid and kidney tissue, gave only the brain specific " B " line.

The confluence of the precipitin line given by the neoplastic tissue with the " B " line of normal brain tissue signifies the presence of brain specific organ antigen in the neoplasm (Fig. 4).

In Table II the findings in 20 neoplasms of neural origin, 4 meningiomas, and 3 neoplasms of the nervous system of metastatic origin, are shown. The presence of brain specific antigen is recorded as a positive result.

TABLE I

| Composition of replacement medium | Cytotoxicity effects observed within 2 hours: Fibroblasts | Neoplastic cells |
|---|---|---|
| Control 1<br>Nutritive medium only. (Medium 199 with 20% horse serum and antibiotics) | – | – |
| Experimental<br>Nutritive medium containing 1 in 5 dilution of immune serum and 1 in 10 dilution of complement | – | + |
| Control 2<br>Nutritive medium containing 1 in 5 dilution of immune serum | – | –* |
| Control 3<br>Nutritive medium containing 1 in 10 dilution of complement | – | – |
| Control 4<br>Nutritive medium containing 1 in 5 dilution of normal rabbit serum and 1 in 10 dilution of complement | – | – |

+ = Presence of cytotoxic effect.
– = Absence of cytotoxic effect.
* = Subsequent addition of complement (guinea-pig serum) resulted in a cytotoxic effect within 10 minutes.

*Cytotoxic effect of antiserum on living tissue*

Our observations on normal living brain tissue in tissue cultures have shown that antiserum adequately absorbed with mixed homogenate of liver, kidney and thyroid to removed non-specific antibodies, caused a specific complement dependent cytotoxic effect on astrocytes, oligodendrocytes and myelin (Wickremesinghe and Yates, 1971). In the present study, such an absorbed antiserum, tested for its specific effect on normal glial cells and myelin in culture, was used for observations on its effect on neoplastic tissue. Several explant cultures from each neoplasm were used to make experimental and control observations. A typical record of the observations is given in Table I.

The results of a typical observation are illustrated in Fig. 5.

The observations on normal tissues (with adequate controls) have shown that the cytotoxic effect was tissue-specific: that is, it showed the presence of an immunologically specific material in astrocytes, oligodendrocytes and myelin

(Wickremesinghe and Yates, 1971). Table II shows the results of observations on the cytotoxic effect of the absorbed antiserum on 16 neoplasms of neural origin, 4 meningiomas and 2 metastatic neoplasms of the nervous system. The presence of the immunologically specific material is recorded as a positive result.

*Localization of antigen using fluorescent antiglobulin*

The observations on fluorescent antiglobulin labelling of normal brain tissue exposed to antiserum, showed that specific differential staining of perikarya of astrocytes and oligodendrocytes could be observed using antiserum absorbed with a mixed tissue homogenate of liver, kidney and thyroid, and subsequently diluted (1 part to 2 parts of phosphate buffered saline) before use (Wickremesinghe and Yates, 1971). Although non-specific staining occurred, we have found that,

TABLE II

| Histological diagnosis | Number of cases | Gel diffusion | Immunocyto-toxicity | Immunofluorescence (a) Cryostat | (b) Cells in explant cultures |
|---|---|---|---|---|---|
| Benign astrocytoma | 3 | + | + | + | + |
| Anaplastic astrocytoma | 1 | — | — | — | — |
| Astroblastoma | 1 | — | + | ± | ○ |
| Polar spongioblastoma | 1 | ○ | ○ | ± | ○ |
| Oligodendroglioma | 2 | + | + | + | + |
| Ependymoma | 1 | + | ○ | ○ | ○ |
| | 1 | — | — | — | — |
| Glioblastoma | 4 | — | — | — | — |
| | 1 | — | + | ± | ± |
| Medulloblastoma | 1 | — | ○ | ○ | ○ |
| Neuroblastoma | 1 | — | — | — | — |
| Schwannoma | 2 | — | — | ± | ○ |
| Neurofibroma | 2 | — | — | ○ | ○ |
| Meningioma | 4 | — | — | — | — |
| Secondary carcinoma | 2 | — | — | — | — |
| | 1 | — | ○ | ○ | ○ |

± = Positive result, but staining of cells was not uniformly present.
— = Negative result.
+ = Fluorescent.
○ = Investigation not carried out.

using the techniques we adopted, staining of perikarya in tissue sections by absorbed antiserum, signified immuno-specificity (Fig. 6 and 7). We are less certain of the significance of the staining of cells in explant tissue culture, but there has been a general concurrence of results.

Fig. 8, 9, 10 and 11 are illustrative of the observations made on localization of the specific antigenic material in neoplastic cells.

The sites of fluorescein labelling of neoplastic cells in explant culture varied. In some, there was staining of cell processes or cell membranes, but not perikarya. In others, only the perikarya were stained. Yet others showed irregular staining. Uniform and intense staining of perikarya and processes was observed in the oligodendrogliomas (*cf.* Fig. 9, 10 and 11).

Table II shows the results of these immunofluorescence studies on neoplastic tissue (17 neoplasms of neural origin, 4 meningiomas and 2 metastatic neoplasms of the nervous system).

### DISCUSSION

The loss of organ specific antigens has been reported in experimental rat-liver neoplasms (Weiler, 1959; Nairn, Richmond, McEntegart and Fothergill, 1960; Hiramoto, Bernecky, Jurandowski and Pressman, 1961); in stilboestrol-induced renal carcinoma in the hamster (Weiler, 1956; Nairn *et al.*, 1960); in naturally occurring malignant human skin neoplasms (Nairn *et al.*, 1960); in human testicular neoplasms (Hiramoto, Jurand, Bernecky and Pressman, 1962), and malignant gastro-intestinal neoplasms (Nairn, Fothergill, McEntegart and Richmond, 1962); and in a variety of non-neural neoplasms by Tee, Wang and Watkins (1964). On the other hand the specific antigen was found in benign intestinal polyps (Nairn *et al.*, 1962).

A correlation of the results obtained by three methods for detecting organ-specific antigenic material in tumours of the nervous system adopted in the present study, is given in Table II.

The tissue specific antigenic material identified in astrocytes and oligodendrocytes of normal tissue was detected in the cells of 7 gliomas of astrocytic and oligodendrocytic histogenesis. Four of these were histologically benign (Kernohan Grade 1) neoplasia, and 3 were Grade 2 neoplasia. A polar spongioblastoma of the type defined by Russell and Rubinstein (1963) and a single glioblastoma (Grade 3) also had the specific material in scattered groups of cells. Of two benign ependymomas, one showed the presence of glia-specific antigen and one did not. The former was of the gliovascular type, and the latter of the epithelial type.

The glia-specific antigen was not demonstrable in an anaplastic astrocytoma and 4 glioblastomas (Grade 4), and a medulloblastoma. Nor was the material detected in a neuroblastoma and 11 neoplasia not of glial origin, which included meningiomas, schwannomas, neurofibromas and secondary carcinomas.

Considering the results as a whole, we conclude that the glia-specific antigenic material is present in the benign and less anaplastic neoplastic cells of glial histogenesis; that it is absent from the highly anaplastic glial cells; and that there are qualitative and quantitative differences between normal and neoplastic cells in the distribution of the specific antigen in cell membranes.

### *Significance of the loss of organ specific antigens from neoplastic cells*

Hughes, Louis, Dineen and Spector (1957) and King, Hughes and Louis (1958) have shown that the immunofluorescent staining of normal tissue and non-staining of malignant neoplastic tissue can be observed even after treatment with non-immune sera. Consequently the fact that specific immune sera cause normal tissues to be stained, but not malignant neoplastic tissues, cannot be interpreted as being due to loss of a specific antigenic material. Lycette and Leslie (1965) have shown differential auto-fluorescence between untreated normal and malignant neoplastic tissues.

Nairn *et al.* (1960) and Hiramoto *et al.* (1961) have shown that by absorption of sera and the use of suitable controls it is possible to distinguish between specific immunological staining and non-specific staining.

We have confirmed the presence of an immunologically specific material in normal glial cells (Wickremesinghe and Yates, 1971) and the histologically benign gliomas using three immunological techniques namely gel diffusion, immunocytotoxicity, and immunofluorescence.

It cannot be denied that the non-reactivity of malignant tissue exposed to antiserum may be due to alteration in cell constituents which interfere with the normal combination of antibody with the specific antigenic sites, rather than a loss of a specific antigenic material. Furth (1959) points out that what is behind an antigenic deletion is not necessarily a lack of substance, but a change in a macromolecule.

Alterations in proteins which affect their ability to bind dyes have been demonstrated in a variety of neoplasms by Sorof and Cohen (1951) and Miller and Miller (1952). To determine conclusively that the loss of reactivity to antisera has an immunological basis, one would have to determine whether or not that neoplastic tissue can provoke the formation of specific antibodies capable of blocking the antigenic sites of normal tissue.

More relevant than such a pertinacious investigation into the loss of reactivity to antisera, is the significance of the loss in terms of cell function and activity. Gorer (1961) points out that " loss of iso-antigens is extremely common . . . but it is impossible to say what the significance of the loss might be in terms of cellular behaviour ". Our investigations have shown that where the specific antigenic material is not demonstrable in the cell membrane of neoplastic glial cells, there is a loss of reactivity to cytotoxic antisera, and alterations in the activity and structure of cell membranes of living cells in tissue culture (Wickremesinghe and Yates, 1971, not yet published).

The mitosis inhibiting tissue specific material (chalone) demonstrated by Bullough and Laurence (1964), Bullough (1964) and Bullough and Rytoma (1965) in several tissues (and perhaps present universally) being a complex protein, would be antigenic, and should therefore be a constituent of antigenic tissue specific material demonstrated immunologically. It can therefore be anticipated that loss of chalone activity would be another accompaniment of the loss of tissue specific antigen. Vogt (1958) has shown that tissue specific antigen is located in the membranes of endoplasmic reticulum, cell organelles and cell surfaces. Our observations corroborate its localization in cell membranes. Since cell membranes such as endoplasmic reticulum are the site of fundamental metabolic activities which characterize a living cell, it is not surprising that there is an association between tissue specific antigen and cell form and function.

Green (1958*a*, 1958*b*, 1959) has suggested that an immunological reaction induced by a combination of carcinogen with tissue specific antigen leads to loss of tissue specific antigen from some cells, as an adaptive change, and that these cells, not being subject to growth control mechanisms because of the loss of antigen, give rise to neoplasia.

The term " tissue specific antigen " in this context can be a source of confusion. An antigen is a substance capable of forming an antibody, so that a tissue specific antigen merely signifies the presence of some substance which is peculiar to that tissue, and which can provoke the formation of antibody. So the loss of growth control which is attributed to loss of antigen is really not due to loss of antigenicity *per se*, but to a loss of some material which has the property of growth control. The fact that this material is antigenic permits the demonstration of its presence or absence by immunological methods. Gorer (1961) has commented that the demonstration of the loss of a hypothetical growth controlling material immunologically, would not justify the elaboration of an immunological theory of carcinogenesis—any more than an electrophoretic theory of carcinogenesis could be justified if the loss is demonstrated electrophoretically. Green has however proposed that an auto-immune reaction may be responsible for the loss of the hypothetical growth controlling substance. Bullough and Laurence (1964) and Bullough (1964) have demonstrated a property of tissue specific material that could be involved in growth control, namely the ability to inhibit mitosis. But it must be emphasized that growth control encompasses not only the control of size and mitotic rate of cells, but also their differentiation, organization and morphogenesis.

In conclusion, it can be said that, since antigen-antibody reactions are due to structural reciprocity at macro-molecular level, it is a structural alteration of macro-molecules, that is, anaplasia at macro-molecular level, which is recognized as "loss of tissue specific antigen ". The significance of this alteration could be assessed if observations on cellular behaviour were carefully correlated with the immunological investigations.

We are grateful for permission to use Fig. 1, 2, 3, 6 and 7 which were previously published in our article in the " Journal of the Neurological Sciences ".

### REFERENCES

BULLOUGH, W. S.—(1964) ' Growth regulation by tissue-specific factors or chalones '. In: 'Cellular Control Mechanisms and Cancer ', edited by P. Emmelot and O. Muhlbock. Amsterdam (Elsevier Pub. Co.), pp. 124–145.

BULLOUGH, W. S. AND LAURENCE, E. B.—(1964) *Expl Cell Res.*, **33**, 176.

BULLOUGH, W. S. AND RYTOMA, T.—(1965) *Nature, Lond.*, **205**, 573.

ELEK, S. D.—(1948) *Br. med. J.*, i, 493.

FURTH, J.—(1959) ' Loss of specific antigen in relation to carcinogenesis '. In: ' Carcinogenesis: Mechanisms of Action ', edited by G. E. W. Wolstenholme and M. O'Connor. Ciba Foundation Symposium. London (J. & A. Churchill).

GORER, P. A.—(1961) ' The antigenic structure of tumours '. In: ' Advances in Immunology ', edited by W. H. Taliaferro and J. H. Humphrey. N.Y. and London (Academic Press), Vol. 1, pp. 345–399.

GREEN, H. N.—(1958*a*) *J. chron. Dis.*, **8**, 123.—(1958*b*) *Br. med. Bull.*, **14**, 101.—(1959) ' Immunological aspects of cancer '. In: ' Carcinogenesis: Mechanisms of Action ', edited by G. E. W. Wolstenholme and M. O'Connor. Ciba Foundation Symposium. London (J. & A. Churchill).

HIRAMOTO, R., BERNECKY, J., JURANDOWSKI, J. AND PRESSMAN, D.—(1961) *Cancer Res.*, **21**, 1372.

HIRAMOTO, R., JURAND, J., BERNECKY, J. AND PRESSMAN, D.—(1962) *Proc. Soc. exp. Biol. Med.*, **3**, 505.

HUGHES, P. E., LOUIS, C. J., DINEEN, J. D. AND SPECTOR, W. G.—(1957) *Nature, Lond.*, **180**, 289.

KAPLAN, M. H.—(1958) *J. Immun.*, **80**, 254.

KING, E. S. J., HUGHES, P. E. AND LOUIS, C. J.—(1958) *Br. J. Cancer*, **12**, 5.

LEIGHTON, J.—(1954) *J. natn. Cancer Inst.*, **15**, 275.

LYCETTE, R. M. AND LESLIE, R. B.—(1965) *Lancet*, ii, 436.

MAYERSBACH, H.—(1959) *J. Histochem. Cytochem.*, **7**, 427.

MILLER, E. C. AND MILLER, J. A.—(1952) *Cancer Res.*, **12**, 547.

MORGAN, J. F., MERTON, H. J. AND PARKER, R. C.—(1950) *Proc. Soc. exp. Biol. Med.*, **73**, 1.

NAIRN, R. C. (editor)—(1964) ' Immunological tracing; general considerations '. In: ' Fluorescent Protein Tracing '. London (E. & S. Livingstone)

NAIRN, R. C., FOTHERGILL, J. E., MCENTEGART, M. G. AND RICHMOND, H. G.—(1962) *Br. med. J.*, i, 1791.

NAIRN, R. C., RICHMOND, H. G., MCENTEGART, M. G. AND FOTHERGILL, J. E.—(1960) *Br. med. J.*, i, 1335.

OUCHTERLONY, O.—(1948) *Acta path. microbiol. scand.*, **25**, 186.—(1964) ' Gel-diffusion techniques '. In: ' Immunological Methods. A Symposium ', edited by J. F. Ackroyd. Oxford (Blackwell), pp. 55–78.

RUSSELL, D. S. AND RUBINSTEIN, L. J.—(1963) ' Pathology of Tumours of the Nervous System '. London (Edward Arnold).

SOROF, S. AND COHEN, P. P.—(1951) *Cancer Res.*, **11**, 376.

TEE, D. E., WANG, M. AND WATKINS, J.—(1964) *Nature, Lond.*, **204**, 682.

VOGT, P.—(1958) *Nature, Lond.*, **182**, 1807.

WEILER, E.—(1956) *Br. J. Cancer*, **10**, 560.—(1959) ' Loss of specific cell antigen in relation to carcinogenesis '. In: ' Carcinogenesis: Mechanisms of Action ', edited by G. E. W. Wolstenholme and M. O'Connor. Ciba Foundation Symposium. London (J. & A. Churchill), pp. 165–175.

WELLER, T. H. AND COONS, A. H.—(1954) *Proc. Soc. exp. Biol. Med.*, **86**, 789.

WICKREMESINGHE, H. R. AND YATES, P. O.—(1971) *J. neurol. Sci.* (in press).


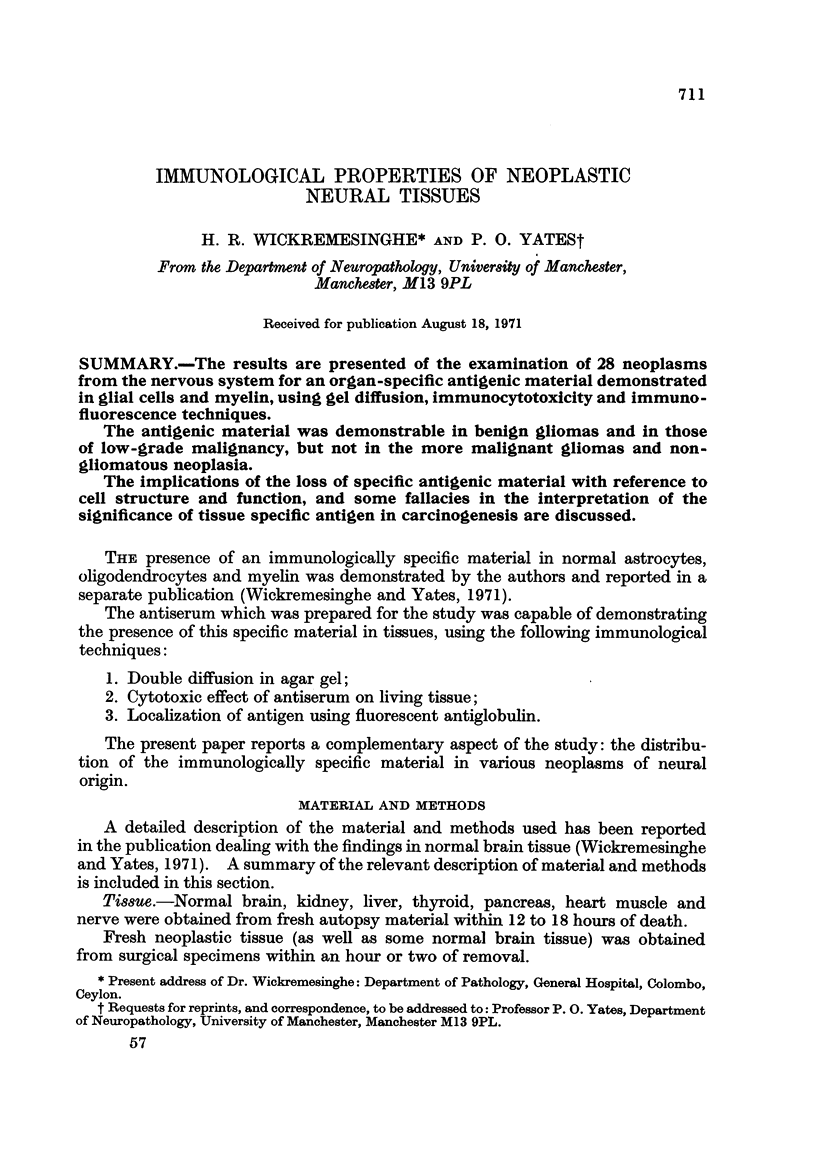

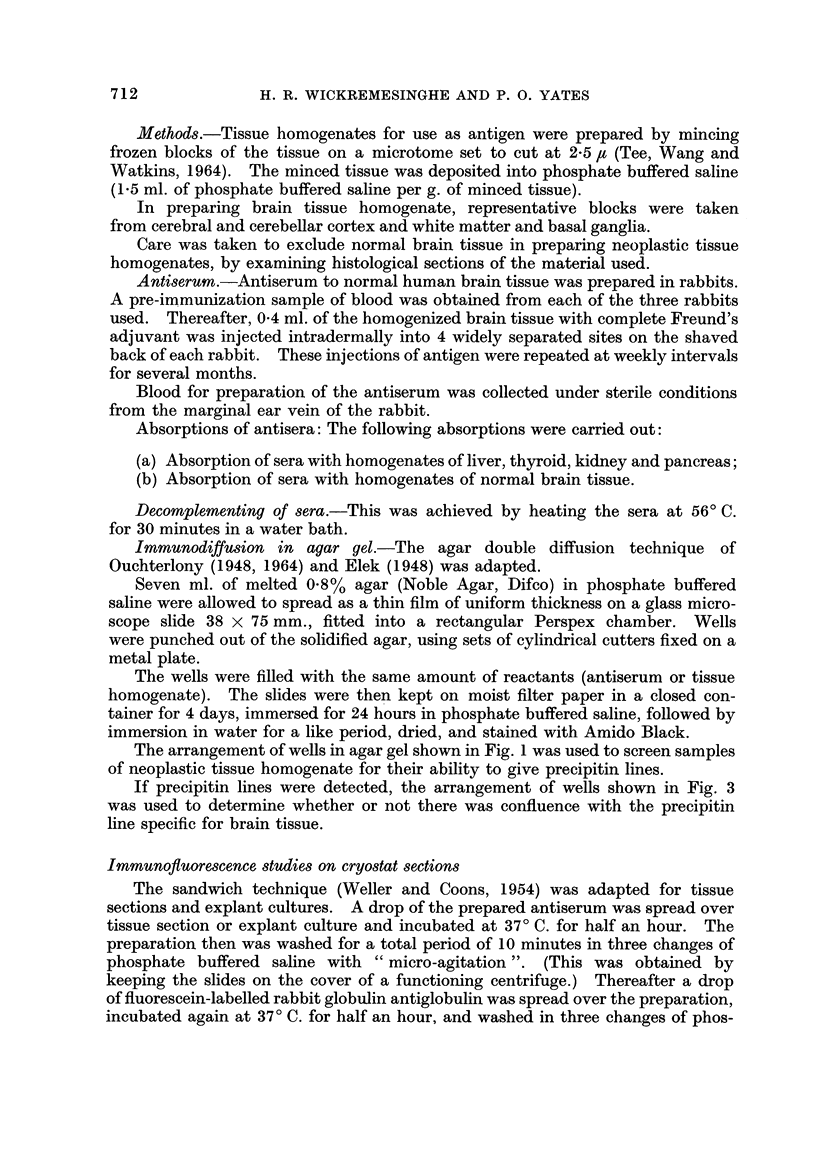

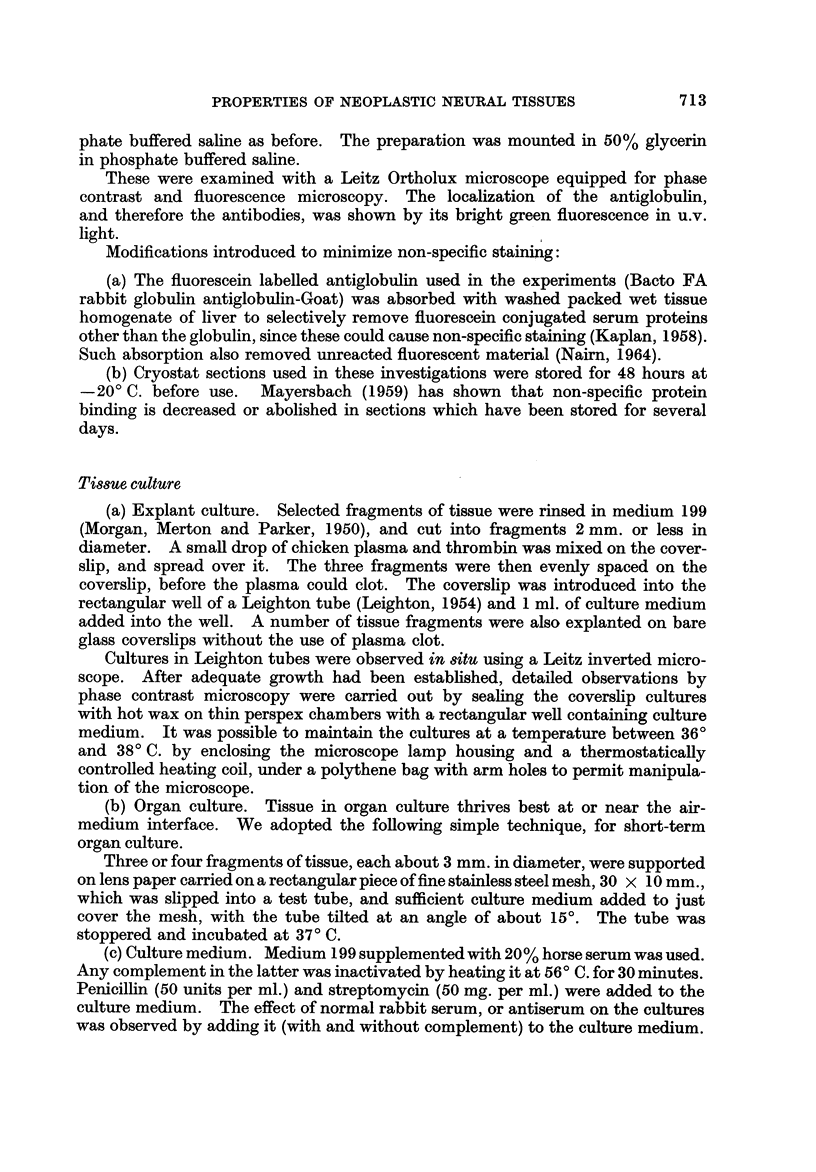

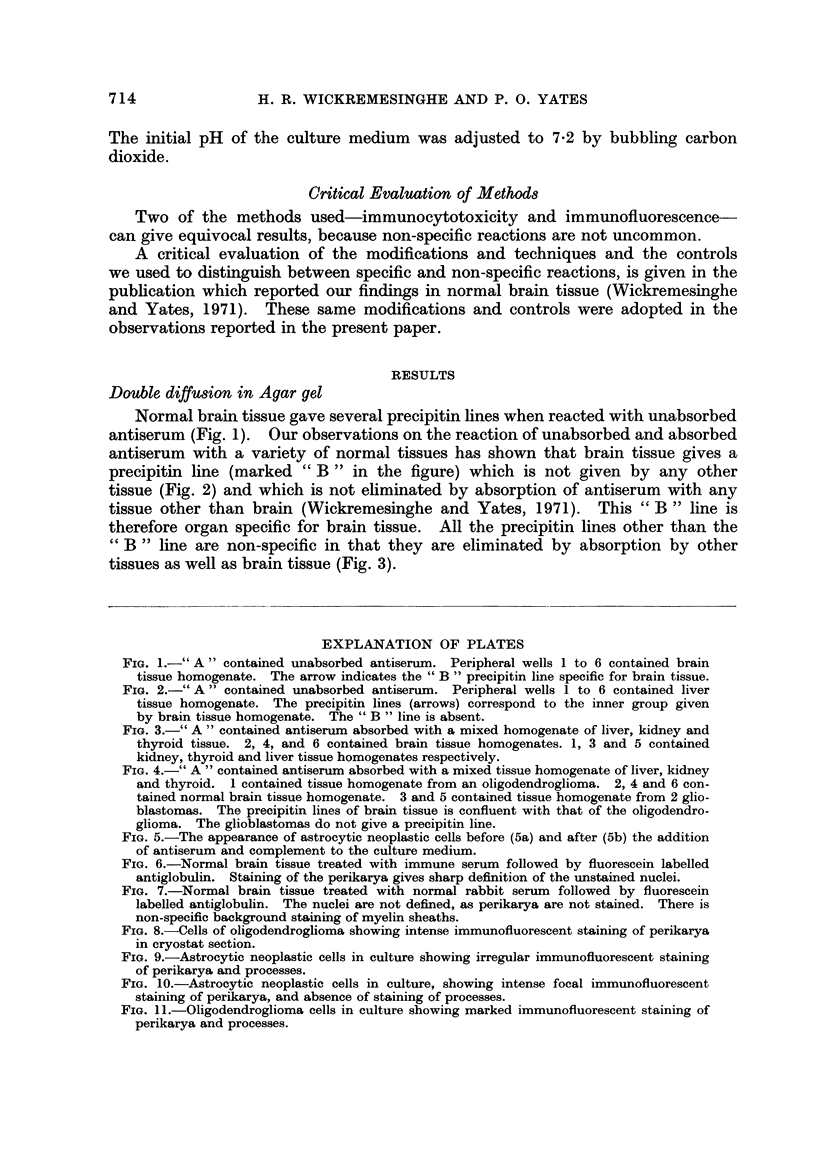

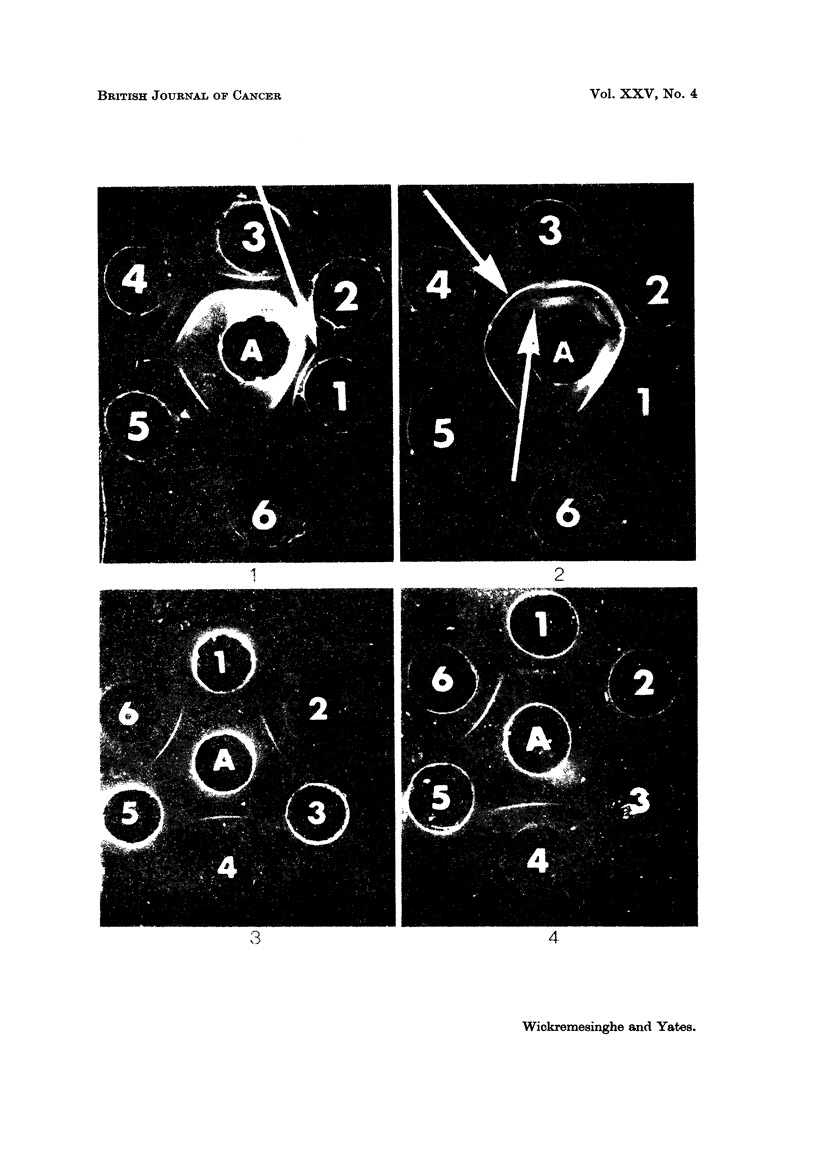

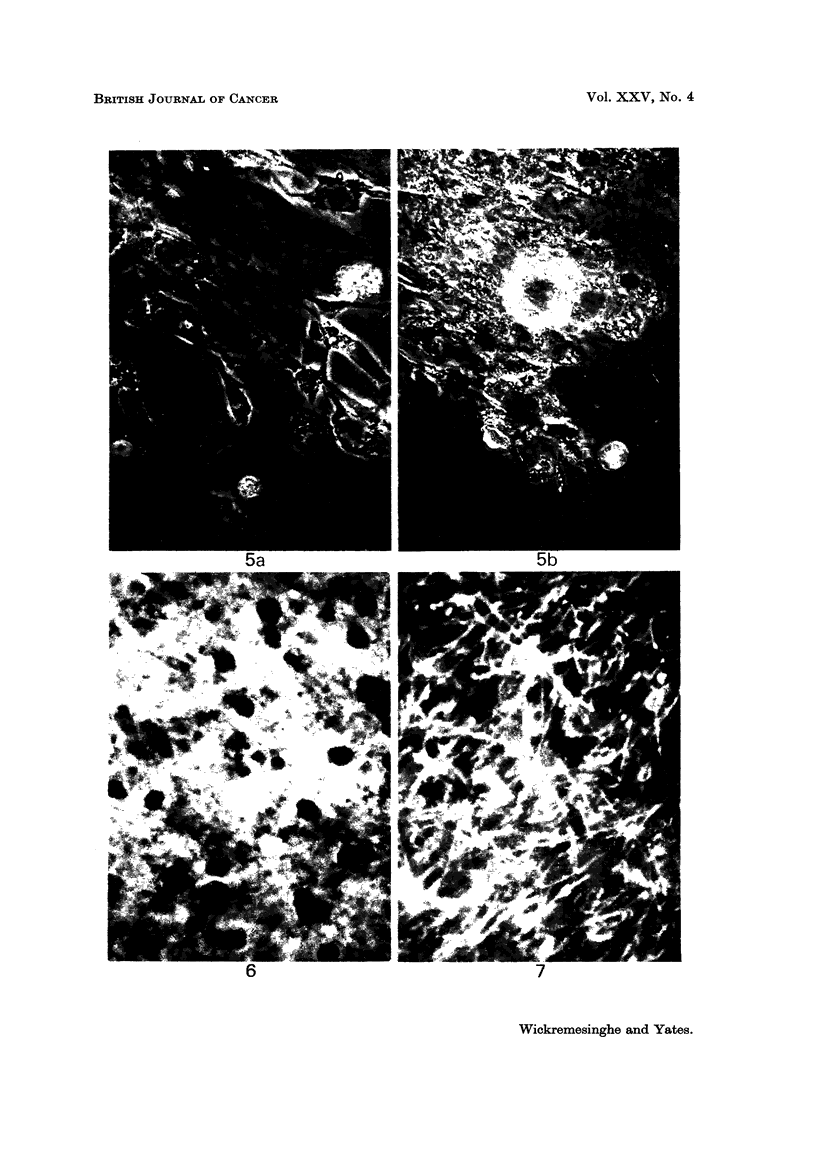

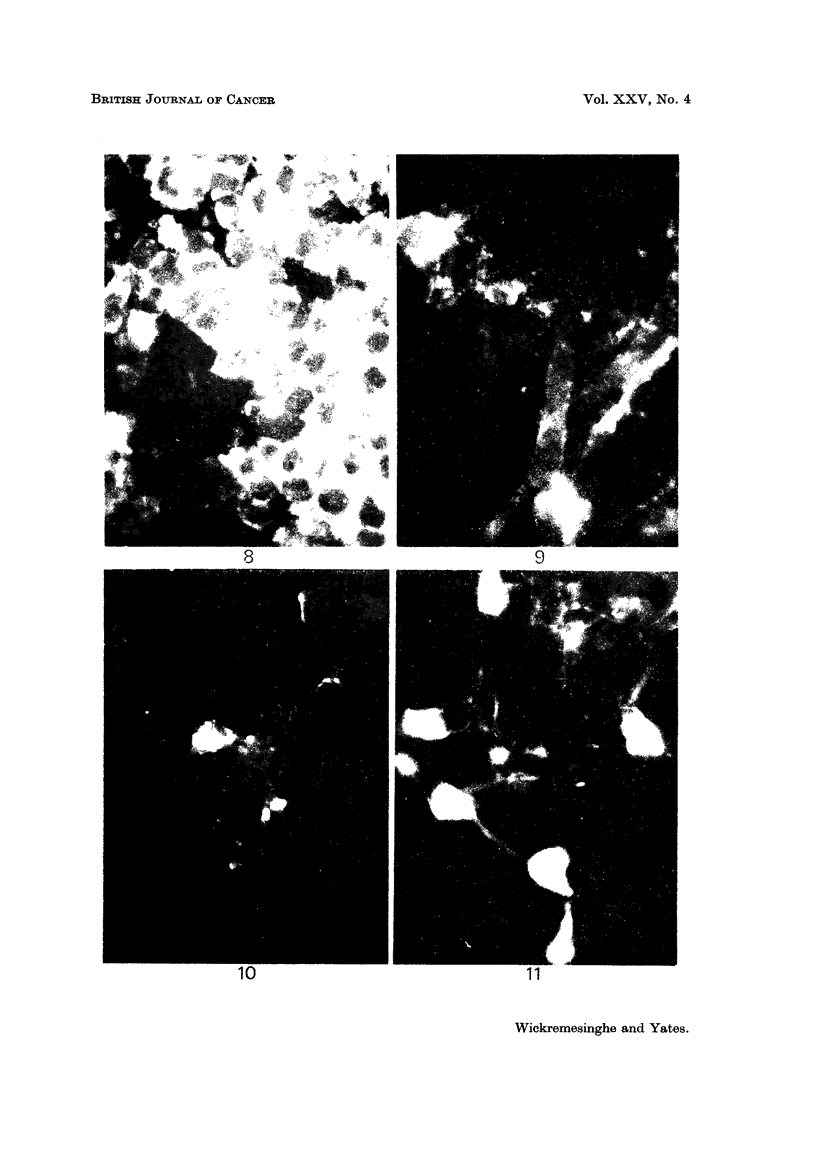

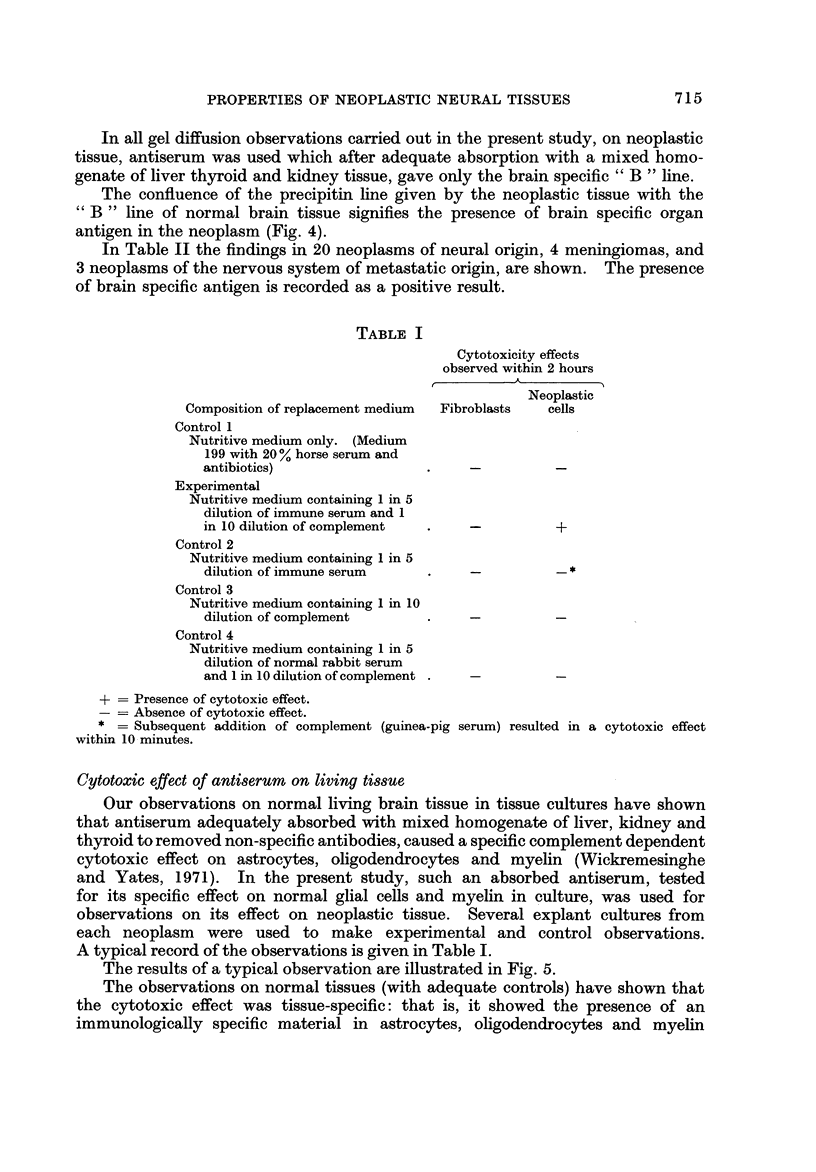

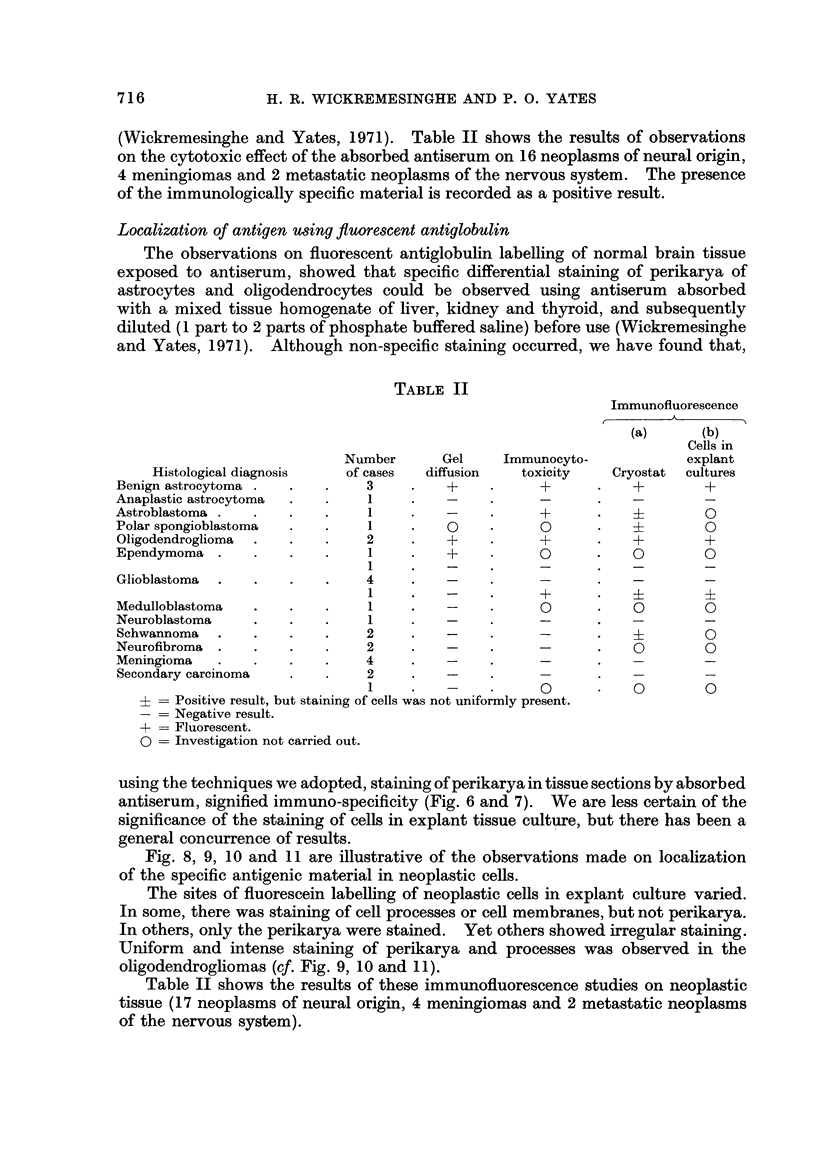

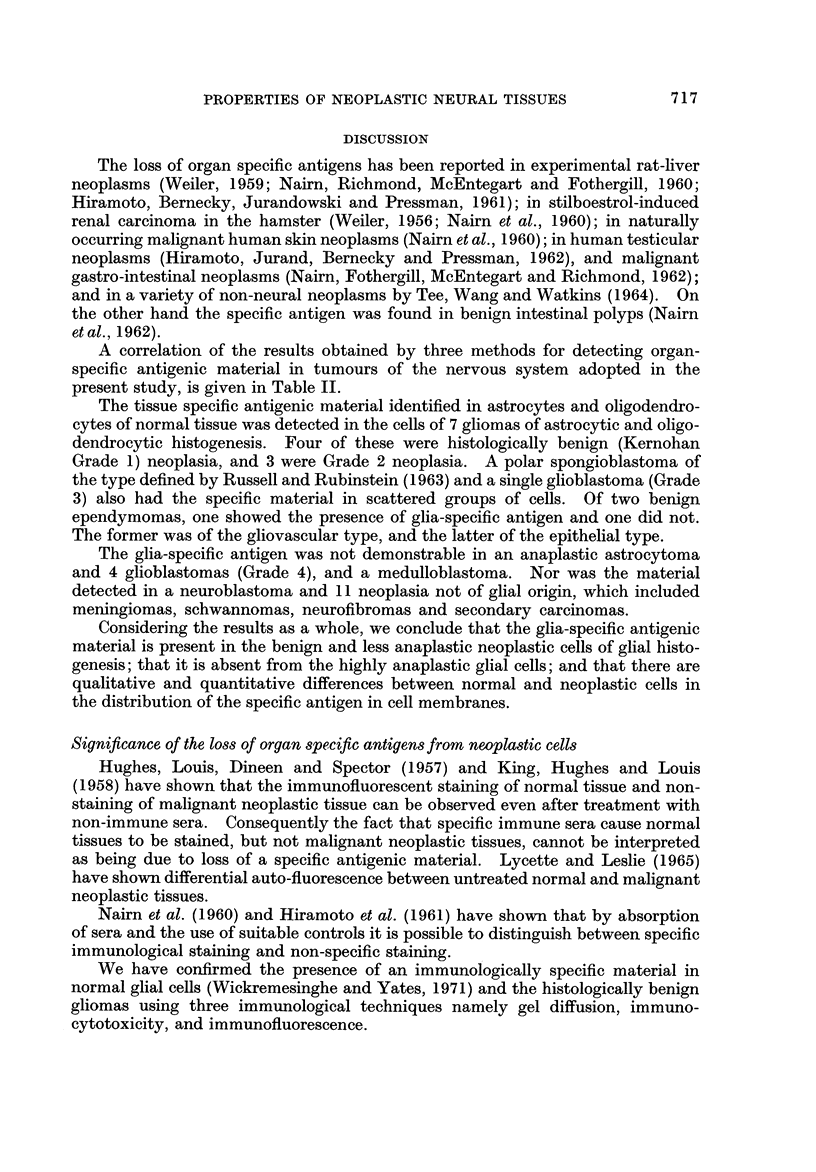

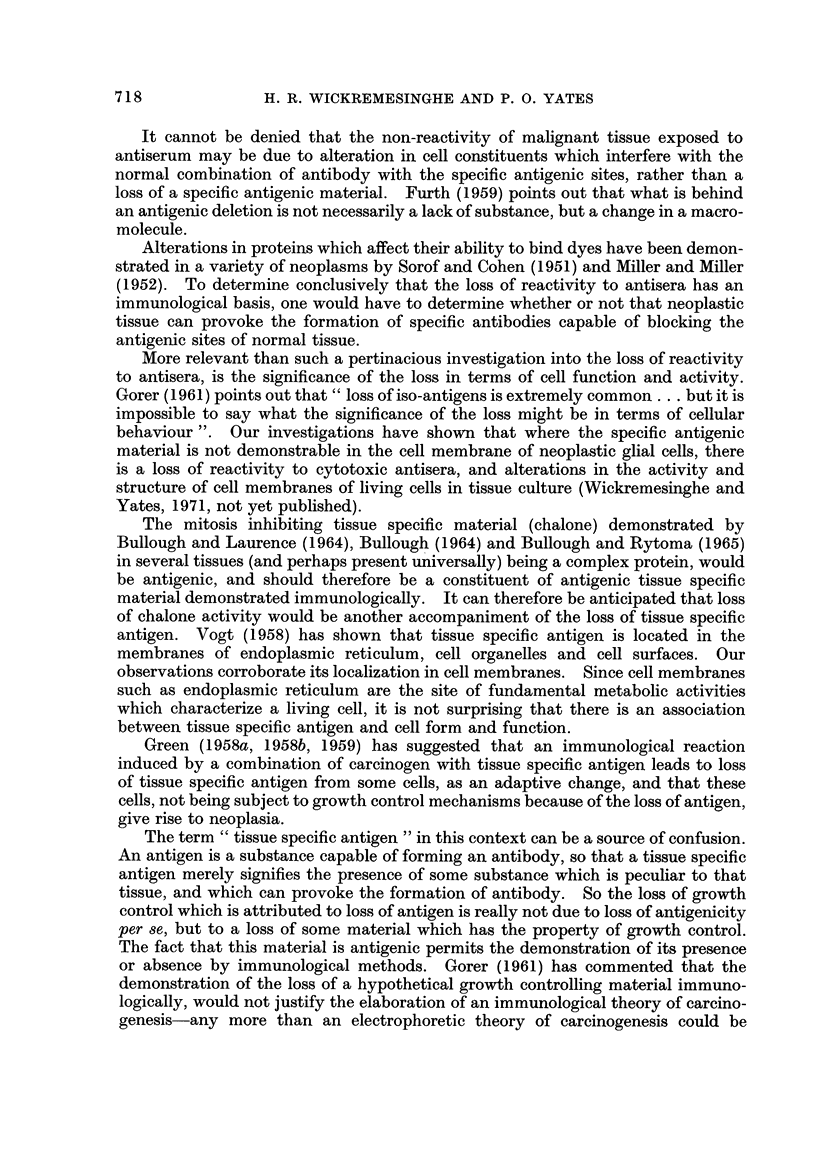

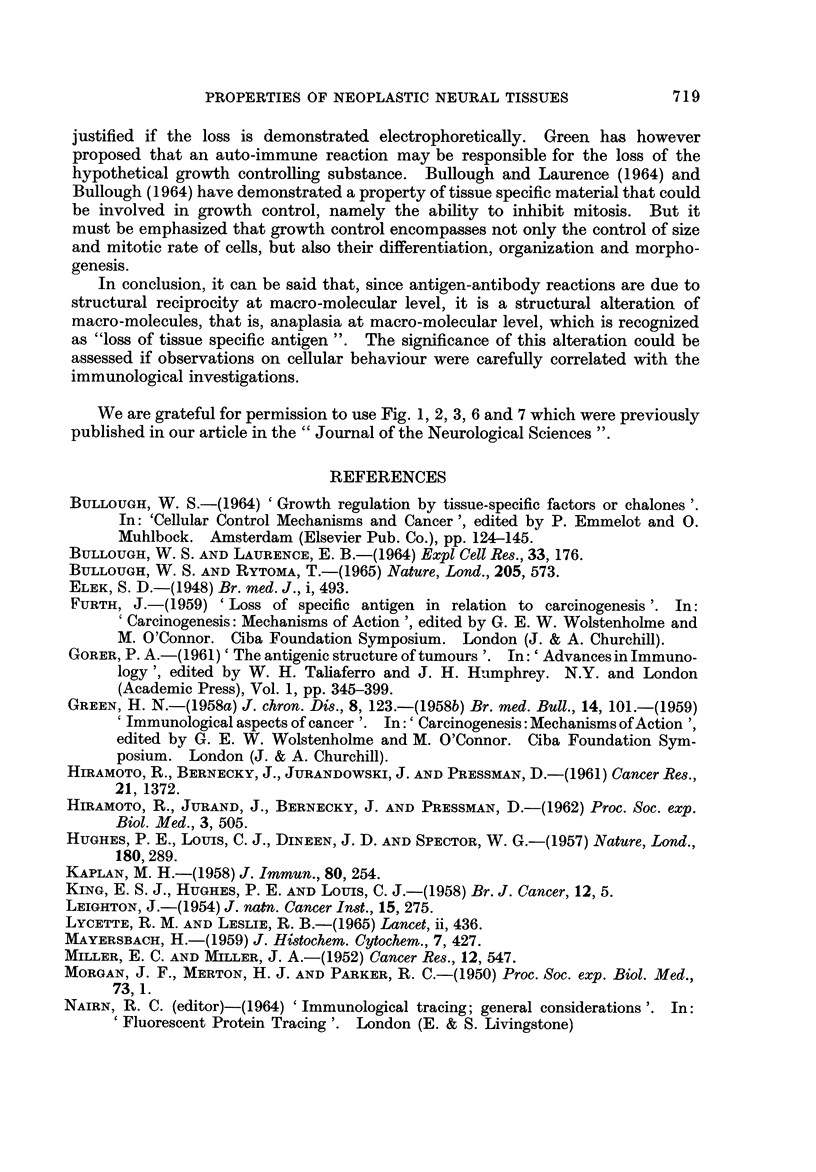

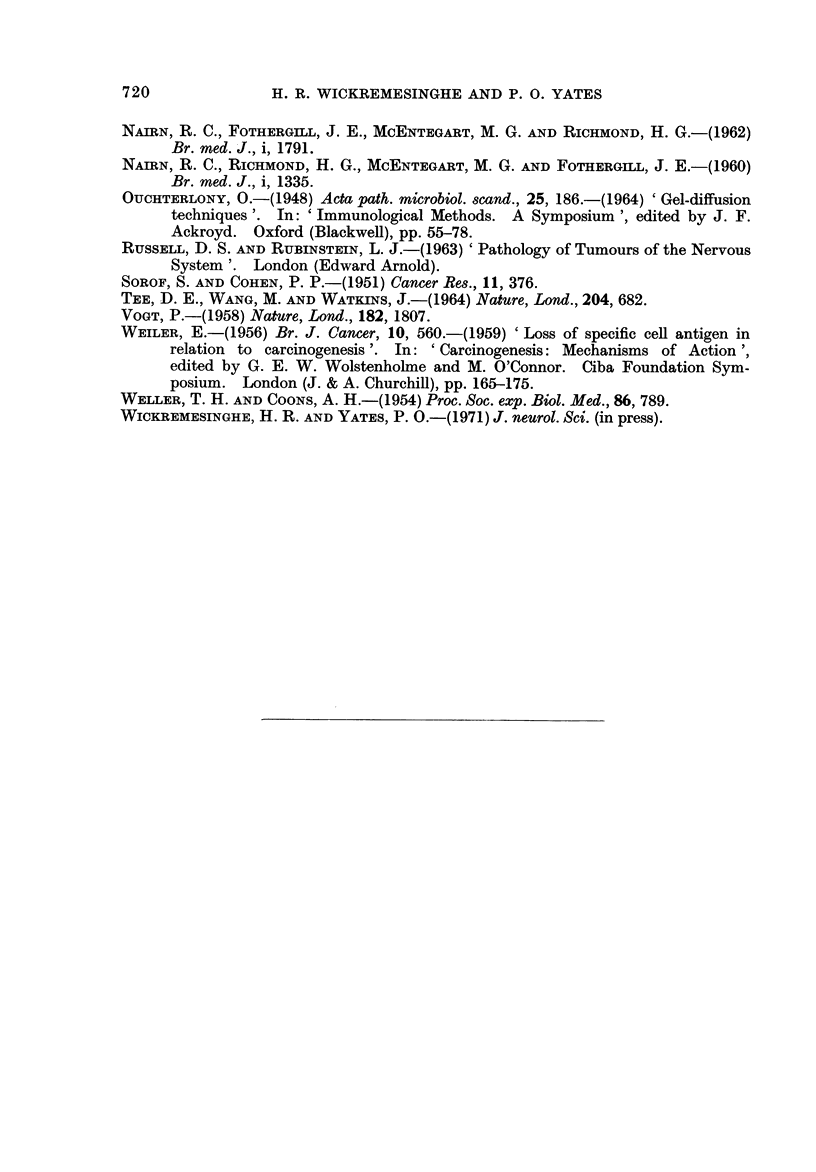


## References

[OCR_00676] HIRAMOTO R., BERNECKY J., JURANDOWSKI J., PRESSMAN D. (1961). Immunohistochemical staining properties of the N-2-FAA rat hepatoma.. Cancer Res.

[OCR_00678] HIRAMOTO R., JURAND J., BERNECKY J., PRESSMAN D. (1962). Lack of staining of testicular tumors by anti-sperm and anti-testis antibodies.. Proc Soc Exp Biol Med.

[OCR_00682] HUGHES P. E., LOUIS C. J., DINEEN J. K., SPECTOR W. G. (1957). Role of organ-specific antigen during 4-dimethylaminoazobenzene carcinogenesis in the rat liver.. Nature.

[OCR_00692] MAYERSBACH H. (1959). Unspecific interactions between serum and tissue sections in the fluorescent-antibody technic for tracing antigens in tissues.. J Histochem Cytochem.

[OCR_00712] NAIRN R. C., RICHMOND H. G., McENTEGART M. G., FOTHERGILL J. E. (1960). Immunological differences between normal and malignant cells.. Br Med J.

[OCR_00706] Nairn R. C., Fothergill J. E., McEntegart M. G., Richmond H. G. (1962). Loss of Gastro-intestinal-specific Antigen in Neoplasia.. Br Med J.

[OCR_00723] SOROF S., COHEN P. P. (1951). Electrophoretic and ultracentrifugal studies on the soluble proteins of various tumors and of livers from rats fed 4-dimethylaminoazobenzene.. Cancer Res.

[OCR_00725] TEE E. H., WANG M., WATKINS J. (1964). A CRYOSTAT METHOD FOR THE EXTRACTION OF SOLUBLE TISSUE COMPONENTS.. Nature.

[OCR_00726] VOGT P. (1958). Distribution of tissue-specific antigens in centrifugal fractions of rat liver.. Nature.

[OCR_00728] WEILER E. (1956). Antigenic differences between normal hamster kidney and stilboestrol induced kidney carcinoma: histological demonstration by means of fluorescing antibodies.. Br J Cancer.

[OCR_00736] WELLER T. H., COONS A. H. (1954). Fluorescent antibody studies with agents of varicella and herpes zoster propagated in vitro.. Proc Soc Exp Biol Med.

